# A finely resolved phylogeny of Y chromosome Hg J illuminates the processes of Phoenician and Greek colonizations in the Mediterranean

**DOI:** 10.1038/s41598-018-25912-9

**Published:** 2018-05-10

**Authors:** Andrea Finocchio, Beniamino Trombetta, Francesco Messina, Eugenia D’Atanasio, Nejat Akar, Aphrodite Loutradis, Emmanuel I. Michalodimitrakis, Fulvio Cruciani, Andrea Novelletto

**Affiliations:** 1 Department of Biology, University of Rome Tor Vergata, Rome, Italy; 2 Dipartimento di Biologia e Biotecnologie “C. Darwin”, Sapienza Università di Roma, Rome, Italy; 3 Pediatrics Department, TOBB-Economy and Technology University Hospital, Ankara, Turkey; 4 National Center for Thalassemias, Athens, Greece; 5 Department of Forensic Sciences, University of Crete School of Medicine, Heraklion, Crete, Greece; 6 Istituto di Biologia e Patologia Molecolari, CNR, Rome, Italy

## Abstract

In order to improve the phylogeography of the male-specific genetic traces of Greek and Phoenician colonizations on the Northern coasts of the Mediterranean, we performed a geographically structured sampling of seven subclades of haplogroup J in Turkey, Greece and Italy. We resequenced 4.4 Mb of Y-chromosome in 58 subjects, obtaining 1079 high quality variants. We did not find a preferential coalescence of Turkish samples to ancestral nodes, contradicting the simplistic idea of a dispersal and radiation of Hg J as a whole from the Middle East. Upon calibration with an ancient Hg J chromosome, we confirmed that signs of Holocenic Hg J radiations are subtle and date mainly to the Bronze Age. We pinpointed seven variants which could potentially unveil star clusters of sequences, indicative of local expansions. By directly genotyping these variants in Hg J carriers and complementing with published resequenced chromosomes (893 subjects), we provide strong temporal and distributional evidence for markers of the Greek settlement of Magna Graecia (J2a-L397) and Phoenician migrations (rs760148062). Our work generated a minimal but robust list of evolutionarily stable markers to elucidate the demographic dynamics and spatial domains of male-mediated movements across and around the Mediterranean, in the last 6,000 years.

## Introduction

Views on the formation of the European gene pool have recently undergone a remarkable increase in complexity, by virtue of genomic data, new methods of analysis, new correlations with archaeological data, and the genotyping of individuals from different temporal strata. A general conceptual framework is that of multiple genetic layers, sequentially stratified as a result of migrations, admixtures, resettlements and replacements. Furthermore, the impact of each of these processes turns out to differ dramatically in different regional areas of the continent^1–8^. In this context, the contact zones between Europe, Western Asia and Northern Africa have emerged as crucial for the departure, movement and arrival of migrants^9^. The water bodies, too, are now regarded less of a barrier and more of a bridge from the Mesolithic onwards^10–15^. On the background of the pre-Neolithic occupation of Southern Europe, characterized by low population density and strong divergence, coastal and immediate inland areas offered resources for successful settlement and trade, especially for the conveyors of cultural, technological and warfare advances. This may have promoted population growth, admixture with and absorption of pre-existing groups, and more widespread occupation of inhabitable and exploitable territories^16^.

A case study is represented by the Southern half of the Italian peninsula. Its genetic distinctiveness is undisputed, and initially attributed to the Greek founders of Magna Graecia in the 1st millennium BC^17^. Subsequent works^18–24^ reconsidered this hypothesis, leaving the relative contributions by seafarers of the early Neolithic, other inputs in the intervening period, and Greeks of Magna Graecia still unresolved. Yet, Southern Italy offered appropriate conditions for successful settlement, demographic growth and hence molecular radiation. The presence of the expected corresponding signatures can directly be tested by a phylogeographic approach, as applicable to non-recombining portions of the genome. A suitable temporal resolution can be attained, provided that the number of variants is high enough to give a faithful record of a rapid succession of mutational events^25^. This power is not currently attained by massive autosomal SNP data, in which modern patterns of arrangement in a multidimensional space were already established by at least the middle/late Neolithic^1,2^.

The Y chromosome haplogroup J (Hg J) has long been considered the clearest marker of East-to-West migrations that impacted South and South-eastern Europe^26–29^. Our final goal was to determine the source population(s), the relative contribution(s), the routes and the Italian locations where the genetic traces of the above processes are prominent today. This required a deeper understanding of the phyletic affinities between Hg J chromosomes found across the Northern Mediterranean, and of the dynamics of the population(s) to which the Hg J carriers belonged.

We reasoned that lineages ancestral to the Southern Italian (I) ones should be found in one or both of two candidate source populations: Turkey (T) and Greece (G). A topology with Turkish and Greek lineages generally closer than the Italian ones (I,(G,T)), would denote a peculiarity of ancestry in Italy. A mainly (G,(T,I)) topology would be in line with a stronger contribution of Anatolian/Middle Eastern migrants. Finally, a mainly (T,(G,I)) topology would be consistent with a Greek heritage in Southern Italy.

Hence, we used Next Generation Sequencing (NGS) to obtain a large and ascertainment-independent list of variants from 7 subclades within Hg J, each sampled in more than one copy in Turkey, Greece and Italy. This strategy proved successful in providing a first list of evolutionarily stable markers of Greek and Phoenician colonists, but also shed light on demographic processes on a much broader area around the Mediterranean.

## Results

Resequencing of 4.4 Mb of Y chromosome in 58 subjects resulted in approximately 2.7 Mb of high quality positions, of which 1079 turned out to be variable among subjects. In our series, four positions displayed an alternative allele different from that reported in dbSNP (https://www.ncbi.nlm.nih.gov/snp) and two a single alternative allele at an otherwise triallelic position (Supplemental Table 2).

When compared with studies addressing targeted regions or the complete chromosome, a remarkable proportion of our SNPs (625/1079 = 58%) turned out to be novel, despite all four previous studies included a considerable number of Hg J carriers (Supplemental Fig. 5). Also, 642 of our variable positions were not recorded in dbSNP 147 (Supplemental Table 2). Both observations indicate that our sampling scheme captured a poorly known quota of Hg J diversity.

### Features of the Hg J tree

**Figure 1:**
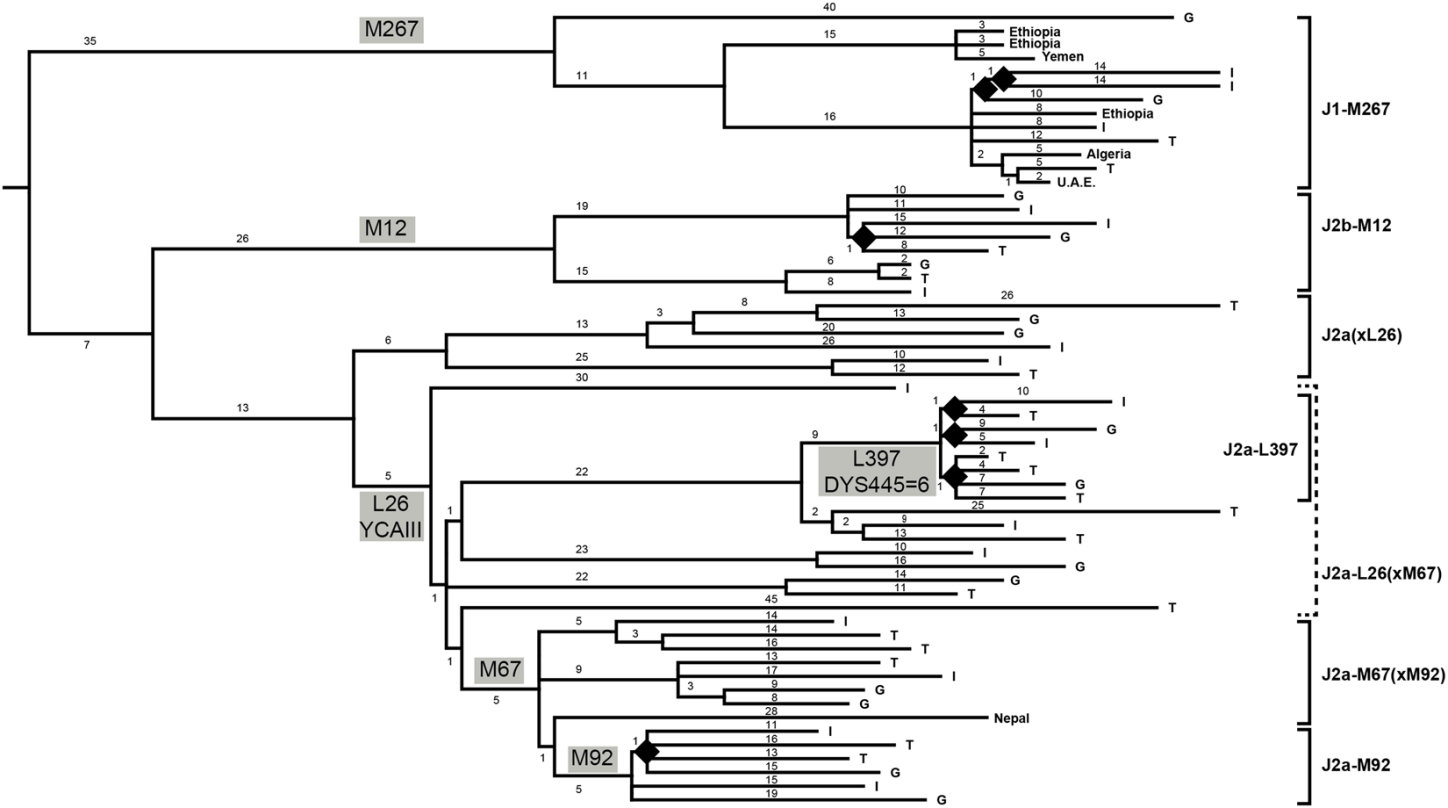
Unrooted MP tree of 58 Hg J chromosomes based on 1079 variable positions (Supplemental Table 2), with the root placed based on the reference sequence. The seven groupings used in the initial selection of samples are shown on the right. The provenance of subjects is abbreviated for Italy (I), Greece (G) and Turkey (T), or in full for other locations. The markers for the definition of clades and selection of samples are shown next to the branches where they occur, in grey boxes. The number of SNPs defining each branch (branch length) is also reported. Black lozenges indicate nodes discussed in the text, for which specific SNP assays were applied.

The topological relationships among the 7 selected subclades in the MP tree (Fig. 1 and Supplemental Fig. 3), recapitulated the known Hg J phylogeny, with a first split into J1 and J2. The internal topology of J1 was strongly influenced by a single deeply rooted sample (branch 1) and a subclade (J1-P56, branch 6) clustering a Yemenite and two Ethiopian subjects. A multifurcation grouped together all remaining subjects sampled as J1-M267 (and found to carry J1-P58) in a vast area spanning from Italy to Africa and the Arabian Peninsula.

Within J2, an early split separated J2a-M410 from J2b-M12. Within this latter, both J2b-M205 (5 subjects) and J2b-M241 (3 subjects) were sampled.

Within J2a-M410, a lineage characterized by the ancestral YCAIII microsatellite pattern with >18 repeats^26^ separated early. In view of the phylogenetic equivalence with L26/Page55, this lineage was indicated also as J2a(xL26).

Among carriers of the derived YCAIII pattern ≤18 repeats, a single subject (FOG05) was found to define a deep branch, while the remaining subjects defined 5 fast radiating lineages. One of these (branch 60) leads to the selected J2a-L397 clade (8 subjects). Subjects selected as carriers of L397 (associated with the 6 repeats allele at DYS445) were further split by 3 variants.

In the clade defined by M67, three sister lineages were sampled, one of which leading to J2a-M92. Finally, within the J2a-M92 clade one variant (branch 98) clustered 4 of the 6 subjects.

The assignment of SNPs to each of the tree branches (Supplemental Table 2) revealed that, as expected, most of the variants not previously annotated belonged to private branches (Supplemental Fig. 3). However, their distribution greatly contributed to the definition of previously poorly known lineages, i.e. 1) a small clade within J1-P58 (branch 18); 2) a small clade within J2b-M205 (branch 29)^30,31^; 3) a clade (branch 41) grouping 4 of the 6 J2a(xL26)^30,32^; 4) three branches within J2a-L397, one of which previously unknown (branch 58); 5) a small clade (branch 62) grouping 3 subjects; 6) a branch within J2a-M92 (branch 98), not reported in any of the previous studies. It is worth noting that all the above small clades grouped subjects from at least 2 of our 3 broad sampling areas. Thus, the low representation of these clades is not due to a geographically insufficient sampling but, rather, to a low frequency despite a large area of occurrence.

Another main aspect of the tree shown in Fig. 1 replicated the peculiarity of Hg J as opposed to other haplogroups in refs^31–36^ (hereafter “previous landmark studies”) and others, i.e. the large variations in branch length and the common occurrence of very deep lineages as compared to the entire haplogroup. Even between sister lineages in sub-terminal branches, large variations in the accumulation of variants were observed. We tested^37^ the equality of branch lengths and obtained non significant departures from the null, either across the entire tree (Supplemental Fig. 2B), or within J2 and J1 (not shown). In addition, seven branches which appeared as private were equal or longer than 25 mutational steps (Fig. 1). All these features were paralleled by the weak evidence for star-like radiations.

Finally, a main topological characteristic of the tree was immediately at odds with the simple idea that the radiation within Hg J occurred during a progressive westward dispersal of people from Turkey (Anatolia) towards the Aegean Islands, Greece and Italy^26–28,38^: when looking at each of the 7 selected subclades or their internal sub-branches, no evidence for a preferential occurrence of the (T,(G,I)) topology emerged, despite the spatially-specific sampling scheme.

### Dating and inferences on demography

We obtained node ages using an ancient specimen sequenced at relatively high coverage, chosen from within Hg J. The calibrated mutation rate fell within the range of values reported in recent studies and obtained with a variety of methods^39,40^. The dates obtained here for some key nodes of the tree (Supplemental Fig. 4) were in close agreement with those of previous studies. Two temporal windows showed an enrichment of nodes, i.e. 16–14 kya and 6-3 kya. Moreover, within J2a(xL26), J2a-L26(xM67), -M67(xM92) and -M92 a paucity of nodes was observed from 5 kya to present. The Bayesian reconstruction returned a population growth rate as low as 1.4% per generation (95% C.I. 0.6–2.9%)^41^.

In order to gain insights on the demography of population(s) of which the Hg J carriers are reporters, we generated Bayesian Skyline Plots for the entire tree and some lineages (Supplemental Fig. 5). The whole tree displayed two phases of growth, at 15 kya and 5 kya onward, respectively. Interestingly, this plot showed a slowdown of growth in the last 2.5 kya, replicating the Greek and Turkish results, which differ from the pattern of at least 12 other European populations^42^.

The above analysis should be considered with caution, as far as the number of chromosomes in each of the 7 selected clades was not necessarily proportional to their frequency in the population. We then repeated the analysis for 5 groups of chromosomes devoid of nested subclades also used in the selection process (e.g. J2a-M92 within J2a-M67). Both J1-M267 and J2b-M12 displayed a sudden increase of population size around 4–5 kya, followed by stasis. J2a(xL26) showed a progressive increase until 5 kya, again followed by stasis. Finally, J2a-L397 and J2a-M92 did not show changes during their respective ages.

### Closing on recent growth episodes

The above results returned the picture of Hg J as a collection of ancient lineages surviving within the population at a very modest growth. The resequencing of 18 chromosomes from Southern Italy, where the impact of the Greeks has been proposed^17^ to dominate the genetic landscape, failed to show the signs of massive growth in any of the selected clades. In these circumstances, we sought to locate more subtle signs of growth, by spotting candidate tree branches and searching subjects falling into them, by means of genotyping. We pinpointed seven variants which could potentially unveil star clusters of sequences (lozenges in Fig. 1). We directly genotyped candidate carriers of these variants and complemented our results with data from resequenced chromosomes (see Materials and Methods).

**Figure 2:**
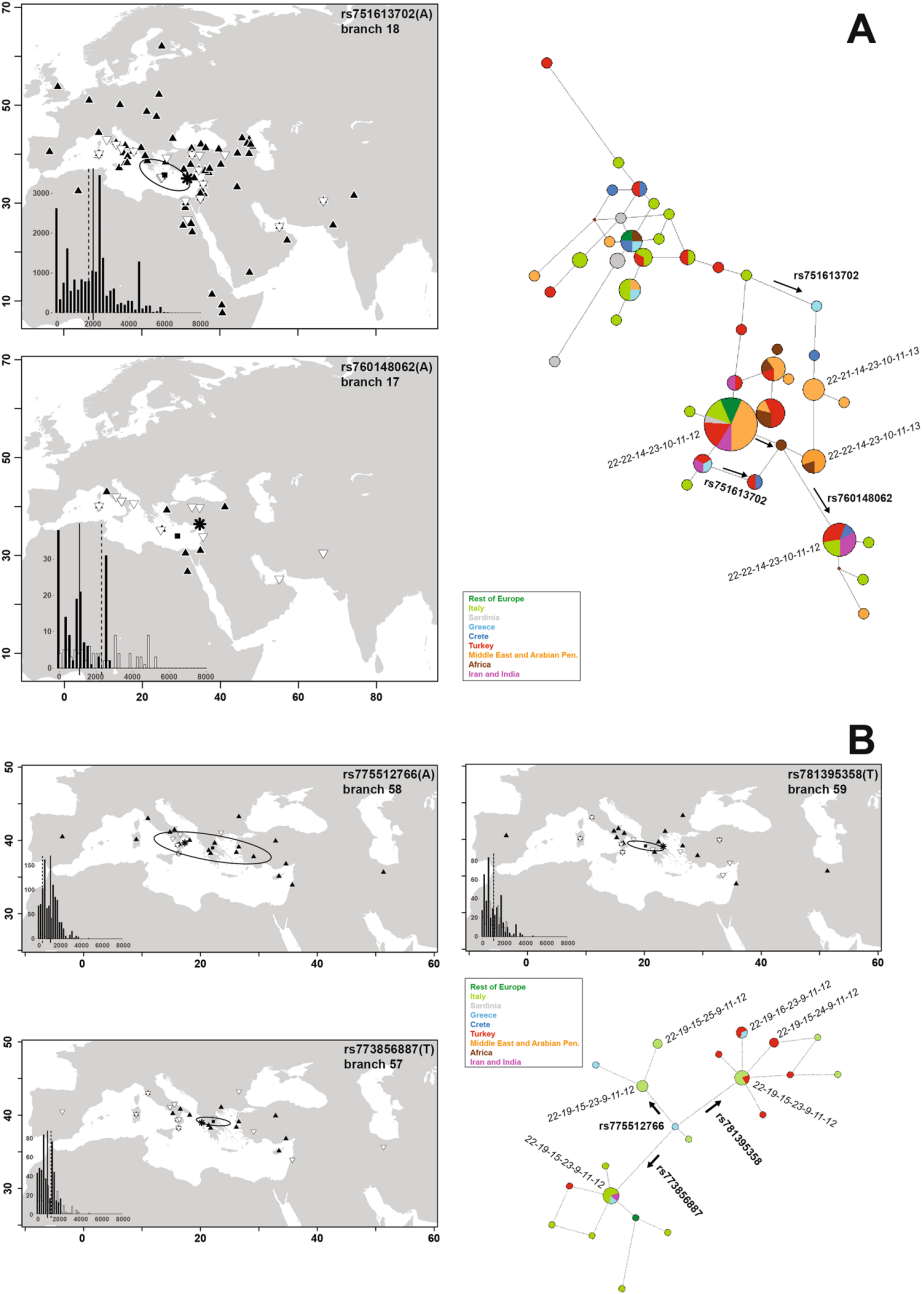
Maps of sampling locations for the carriers of the derived allele (white triangle point down) at the indicated SNP vs carriers of the ancestral allele (black triangle point-up), conditioned on identical genotype at the same most terminal marker. Coastlines were drawn with the R packages “map” and “mapproj” v. 3.1.3 (https://cran.r-project.org/web/packages/mapproj/index.html), and symbols added with default functions. (**A**) J1; (**B**) J2-L397. The star indicates the centroid of derived alleles. The solid square indicates the centroid of ancestral alleles, with its 95% C.I. (ellipse). In the insets: distributions of the pairwise sampling distances (in Km) for the carriers of the ancestral (black) and derived (white) allele, with solid and dashed lines indicating the respective averages. At right: median joining network of 7-STR haplotypes and SNPs in the same groups, with sectors coloured according to sampling location. Haplotype structure is detailed for some nodes, in the order YCA2a-YCA2b-DYS19-DYS390-DYS391-DYS392-DYS393 (in italics).

Within J1-M267 we obtained 32 carriers of the derived allele at rs751613702 (branch 18). They turned out to be sampled at locations (Fig. 2A) as distant as Tuscany (Italy) and Pakistan. Their centroid was significantly shifted to the East as compared to the carriers of the parental lineage (J1-M267). Moreover, the home range of derived alleles was entirely within that of parental lineage, with shorter pairwise distances (t-test p < 1E-4). Further within the same clade, we assayed also rs760148062 (branch 17). We found 14 carriers of the derived allele (Fig. 2A). In this case the distances separating the carriers of the derived allele and their home range denote movements over wider distances than those of the carriers of the ancestral allele (p < 1E-8). In fact 47% (8/17) of the ancestral alleles were confined in the Israeli Bedouins. This difference in migratory history is also witnessed by STRs (Fig. 2A). The large cluster of J1-M267 immediately ancestral to rs751613702 and enriched in Middle Easterners, corresponded to the 14-23-10-11-12 motif at DYS19-390-391-392-393, which matches the haplotypes PCS2+ and PCS4+ providing a Phoenician Colonization Signal^28^. Subjects derived at rs751613702 but ancestral at rs760148062 mostly had DYS19(14)-DYS393(13), i.e. a combination not listed as PCS. Conversely, subjects derived at rs760148062 retained the 14-23-10-11-12 motif.

Within J2b-M12 (Supplemental Fig. 7), we obtained 14 carriers of the derived allele at rs779180992 (branch 29), which resides within J2b-M205. The centroid of the carriers of the parental lineage (J2b-M12) was shifted to the West as compared with the overall sample, in line with the high frequency of this allele in Albania and Western Greece. The carriers of the derived allele at rs779180992 had a centroid significantly shifted to the East, being found in Italy, the Balkan Peninsula, Northern Turkey, Caucasus and in two Middle Easterners, but neither in Sardinia nor Crete. Their sampling distances did not differ (p = n.s.) from the carriers of the ancestral allele. In the STR network, chromosomes derived at rs779180992 did not show any central haplotype, and were variable at all loci but DYS393.

Within J2a-M92 (Supplemental Fig. 7) we genotyped position Y: 8237851 (branch 98). The derived allele was found in 13 subjects of our core sample but in none of the previous landmark studies. The centroid of the carriers of the ancestral alleles was shifted to the West as compared to the overall sample, in line with the enrichment of J2a-M92 in the Western part of our sample^26^. The carriers of the derived allele at Y:8237851 had a centroid significantly shifted to the East, being found in Italy (including Sardinia), but also in Turkey and the Eastern Mediterranean (including Crete and Cyprus). STRs indicated that the mutation at Y:8237851 occurred on a 22-19-14-22-10-11-12 haplotype (YCA2a, YCA2b-DYS19-390-391-392-393), found only in Italy and Sardinia, and radiating in Italy and elsewhere.

Within J2a-L397, we genotyped the three variants defining branches 57, 58 and 59 (Fig. 2B). Only 4 samples were ancestral at all three positions (paragroup J2a-L397*). For each of the three positions, the centroids of carriers of the ancestral allele were located in Greece. The overall distribution of J2a-L397 covered only the northern Mediterranean, with no carriers of this lineage in Cretans of our core sample, but reported at 2.6% by in ref.^43^.

For branch 57 (rs773856887), derived alleles (20) were found at the outer edges of the overall distribution of the carriers of J2a-L397 (p < 1E-7). The STRs network indicated a probable radiation from a 22-19-15-23-9-11-12 haplotype.

The distribution of derived alleles (6) for branch 58 (rs775512766) was strikingly different, with 5 of the 6 instances found in Southern Italy. This concentration resulted in very low pairwise sampling distances (p < 1E-8). Derived alleles were associated with both the 22-19-15-23-9-11-12 and the 22-19-15-25-9-11-12 STR haplotypes. Branches 57 and 58 shared the feature of a centroid for derived alleles shifted to the West as compared to ancestral alleles, though not significantly.

Derived alleles for branch 59 (21) were spread from Italy to Turkey (including Cyprus), covering distances similar, on average, to ancestral alleles (p = n.s.). Here, the STR haplotypes radiated from the founding type to 22-19-16-23-9-11-12 and 22-19-15-24-9-11-12.

Overall, 10/13 of the subjects sampled in a single Italian location (Locri 16.2E, 38.2 N) carried J2a-L397* (as branch 57, 58, or 59), representing 18.5% of all J2a-L397 in the whole sample.

## Discussion

In order to improve our understanding of the patrilinear relationships among populations on the northern coasts of the Mediterranean we addressed specifically Hg J. We used a straightforward spatial representation of sampling locations for a composite sample generated in-house and supplemented with published data. Its composition and distribution is heavily dependent on the particular set of examined populations, on the number of subjects studied for each of them and the local Hg J frequency. We derived our approach from ref.^44^, and calculated the position of centroids and pairwise sampling distances, which are strongly contingent on the sample composition, too. Thus, only comparisons between different partitions of our dataset are meaningful.

Within seven subclades of Hg J we performed a geographically structured sampling and encountered, among others, a number of lineages previously poorly known. We confirmed that J1 contains a number of rare lineages which are diversified and deeply rooted. They have been sampled in Greece, Turkey, the Caucasus and North of it^30,31^ and, surprisingly repeatedly, in America^31,34^. The emergence of these lineages was synchronous (approx. 15 kya) with a similar burst within J2, where several lineages radiated within J2a-M410.

Two features of our tree (Fig. 1) are at odds with the simplistic idea of a dispersal of Hg J as a whole from the Middle East towards Greece and Italy and an accompanying radiation^26^. First, there is little evidence of sudden diversification between 15 and 5 kya, a period of likely population increase and pressure for range expansion, due to the Agricultural revolution in the Fertile Crescent. Second, within each subclade, lineages currently sampled in Turkey do not show up as preferentially ancestral. Both findings are replicated and reinforced by examining the previous landmark studies. Our Turkish samples do not coalesce preferentially to ancestral nodes when mapped onto these studies’ trees.

Additional relevant information on the entire Hg J comes from the discontinuous distribution of J2b-M12. The northern fringe of our sample is enriched in the J2b-M241 subclade, which reappears in the gulf of Bengal^38,45^, with low frequencies in the intervening Iraq^46^ and Iran^47^. No J2b-M12 carriers were found among 35 modern Lebanese, as contrasted to one of two ancient specimens from the same region^35^.

In summary, a first conclusion of our sequencing effort and merge with available data is that the phylogeography of Hg J is complex and hardly explained by the presence of a single population harbouring the major lineages at the onset of agriculture and spreading westward. A unifying explanation for all the above inconsistencies could be a centre of initial radiation outside the area here sampled more densely, i.e. the Caucasus and regions North of it, from which different Hg J subclades may have later reached mainland Italy, Greece and Turkey, possibly following different routes and times. Evidence in this direction comes from the distribution of J2a-M410^45,48^ and the early-^49^ or mid-Holocene^50^ southward spread of J1.

We obtained poor evidence for population growth after 5 kya. A mid-Holocene drop in the male effective size was reported^31^, attributed to the rise of farming and the associated cultural and social habits, which may have increased the variance in offspring number both within and between demes. With the caveat of a equalized sampling across clades, we simply observed a stasis in the 15-5 kya time window in the whole tree, followed by a sudden increase at 5 kya, in line with most European populations^42^. Of the internal clades, only J1-M267 and J2b-M12 displayed the same growth phase at approximately 5 kya. In the context of the recent explosive increase in world population size^41,51^, genetically detectable in Y chromosomal studies of all continents^31^ and Europe^42^, our inferences raise the question of whether individual lineages can survive without undergoing a proportional growth. Here, it is to be noted that the signal of growth for a whole population derives from the multiple haplogroups that contribute to its gene pool. Some of them may have undergone expansion elsewhere, reaching the regions where we found them today, mixed with Hg J, only later. Social and economic circumstances can also act against male-mediated expansions (compare with ref.^25^). For example, occupational roles that involve a mainly patrilineal transmission of wealth to a single (usually the eldest) son, and/or active persecution directed towards paternally inherited cultural traits are viable possibilities.

In order to tackle more directly our goal, we focused on some lineages which suggested limited expansions in a context of generalized modest growth in the last 5–6 kya. The most evident expansion within Hg J in this time frame is provided by J1-P58 in the Middle East. Studies on ancient specimens are revealing that Hg J emerged during the Bronze Age in Lebanon and Jordan, with an earlier record in Iran^1,35^. The increase in frequency^46,49^ and radiation of the J1-P58 lineage is evident in the clustering of subjects from Middle East and Western Arabian Peninsula in this clade. Here, a possible structuring within the Arabian Peninsula is indicated by a single subject (UAE443; Supplemental Fig. 3) from the Eastern side, carrying a lineage (rs867592041; Supplemental Table 2) distinct from those common in Lebanon, Saudi Arabia and Yemen^31,35^. Within J1-P58, we identified two nested sublineages whose age frames between 5 and 4.4 kya the partition into a subset (rs751613702derived-rs760148062ancestral) that remained basically resident and another that underwent a strong dispersal (rs760148062derived). The geographic distribution of the latter and the associated STR haplotypes^28^ make this variant a good candidate for Phoenician migrations^52^.

The lineage defined by rs779180992, belonging to J2b-M205, and dated at 4–4.5 kya, has a radically different distribution, with derived alleles in Continental Italy, Greece and Northern Turkey, and two instances in a Palestinian and a Jew. The interpretation of the spread of this lineage is not straightforward. Tentative hypotheses are linked to Southward movements that occurred in the Balkan Peninsula from the Bronze Age^29,53^, through the Roman occupation and later^54^.

The slightly older (5.6–6.3 kya) branch 98 lineage displays a similar trend of a Eastward positioning of derived alleles, with the notable difference of being present in Sardinia, Crete, Cyprus and Northern Egypt. This feature and the low frequency of the parental J2a-M92 lineage in the Balkans^27^ calls for an explanation different from the above.

Finally, we explored the distribution of J2a-L397 and three derived lineages within it. J2a-L397 is tightly associated with a typical DYS445 6-repeat allele. This has been hypothesized as a marker of the Greek colonizations in the Mediterranean^55^, based on its presence in Greek Anatolia and Provence (France), a region with attested Iron Age Greek contribution. All of our chromosomes in this clade were characterized also by DYS391(9), confirming their Anatolian Greek signature. We resolved the J2a-L397 clade to an unprecedented precision, with three internal markers which allow a finer discrimination than STRs. The ages of the three lineages (2.0–3.0 kya) are compatible with the beginning of the Greek colonial period, in the 8th century BCE. The three subclades have different distributions (Fig. 2B), with two (branches 57, 59) found both East and West to Greece, and one only in Italy (branch 58). As to Mediterranean Islands, J2a-L397 was found in Cyprus^56^ and Crete^43^. Its presence as one of the three branches 57–59 will represent an important test. In Italy all three variants were found mainly along the Western coast (18/25), which hosted the preferred Greek trade cities. The finding of all three differentiated lineages in Locri excludes a local founder effect of a single genealogy. Interestingly, an important Greek colony was established in this location, with continuity of human settlement until modern times. The sample composed of the same subjects displayed genetic affinities with Eastern Greece and the Aegean also at autosomal markers^57^. In summary, the distributions of branches 57–59 mirror the variety of the cities of origin and geographic ranges during the phases of the colonization process^58^.

This work revealed that signs of Holocenic Hg J radiations are subtle, but nevertheless concentrated mainly in the Bronze Age, a critical period for the establishment of genetic structure in Europe^25,42^. Clear signs of Phoenician legacy have been linked to J2^28^, but we detect them also in J1. We found a SNP marker of the Phoenician colonization within a star-like cluster which emerged in the Middle East and sent representatives also beyond the present-day Iran (Fig. 2A).

Molecular signs of the Greek expansion are even more subtle. A peculiarity of our Greek markers is that they relate more to the Aegean and are poorly represented in mainland Greece. This is line with the archeologically documented two step process of establishment of Aegean colonies and subsequent outward colonizations. It is possible that the markers arose in Greek Anatolia. However the intrusion of northern peoples in Continental Greece has been all but excluded based on genetic evidence^7^, and may have abated the frequency of these alleles.

We provided here the strongest temporal and distributional evidence so far for markers which can be attributed to the Greek settlement of Magna Graecia. This process cannot be expected to have conveyed only J2a-L397, but other lineages as well: for example E-V13^59^, which left a clear signal across the Mediterranean^28,55^. J2a-L397 in Central-Southern Continental Italy accounts for only 13% (24/182) of Hg J. At a face value this can be taken as a bottom figure for the Greek contribution to the gene pool of the region. Though the true value may be higher than this, it is unlikely that the bulk of diversity has a Greek origin. Under these circumstances it is not surprising that unequivocal genetic traces of the Greek colonists in Continental Italy have gone undetected so far^19^.

Our work generated a minimal but robust list of evolutionarily stable markers to elucidate the demographic dynamics and spatial domains of male-mediated movements across and around the Mediterranean. The search of additional ones will be demanding. In fact, the particular tree topology (long private branches) imposes the search of many variable positions to detect possible geographically-confined groups of samples that could inform on the most recent time of arrival of certain lineages. Nonetheless, the markers explored here appear of great usefulness. First, they can be validated by specifically searching for them in test cases such as Western vs. Eastern Sicily (not sampled here) where distinct Phoenician and Greek cultural and genetic inputs are documented^60^. Other key locations include the North Tunisian coast (Phoenicians), Western Black Sea, North-Eastern Libyan and Mediterranean Iberian coasts (Greeks). Second, they can be directly addressed in available collections of ancient and modern samples never studied at this level of resolution^61^.

## Methods

### The sample

The core sample consisted of 469 males, carriers of Hg J, from collections of the authors, assembled in the 1980’s, 1990’s and 2000’s. The original sampling was performed by colleagues and operators at a number of collaborating Institutions and included the recording of the subject’s place of residence and informed consent. The subject was also asked to report the origin of his parents. Recent immigrants were excluded. Anonymized blood or DNA samples were then received at the corresponding author’s laboratory.

All of these subjects were typed at least for the markers reported in Fig. 1, which define 7 Hg J subclades, 6 of which monophyletic. For each subclade, candidates for the capture-NGS experiment were selected, who satisfied the condition of representing at least all the three broad regions of Italy, Greece and Turkey. Selection considered the amount and quality of DNA, disregarding information on markers other than the six mentioned above. Seven additional individuals entered the experiment, specifically for their provenance (Nepal, Arabian Peninsula and Africa), or a previous assignment to an interesting lineage (J1-P56). Overall, 59 subjects entered the resequencing experiment (Supplemental Table 1).

For further genotyping at phylogeographically interesting markers in the overall sample, 42 additional individuals from the CEPH HGDP panel were also considered, with typing obtained in-house or from the literature^45^.

For data analysis, 375 additional individuals selected from targeted^30^ or whole genome studies^31–36^ were also considered, taking into account duplication of some subjects. The overall sample thus consisted of 893 subjects, distributed as shown in Table 1 and Supplemental Fig. 1.

**Table 1:** Numerosity of the seven Hg J sub-haplogroups considered in this work, partitioned by sampling region.

	J1-M267	J2a-(xL26)	J2a-L397	J2a-L26(xM67)	J2a-M67(xM92)	J2a-M92	J2b-M12	Total
Rest of Europe	14		2	19	7	2	15	**59**
Italy	24	3	24	53	22	24	32	**182**
Sardinia	68	20	4	40	11	11	25	**179**
Greece	4	6	6	11	10	3	11	**51**
Crete	6	2		37	19	4	1	**69**
Turkey	21	7	11	32	17	4	4	**96**
Caucasus	9	1	2	13	5		1	**31**
Middle East and Arabian Peninsula	48	1	4	35	2		4	**94**
Iran, Pakistan, India	7	17	1	26	4	1	17	**73**
NW-Asia				2	1			**3**
Africa	39	4		5	5	1	2	**56**
Total	**240**	**61**	**54**	**273**	**103**	**50**	**112**	**893**

This study received approval by the intramural ethical committee (Comitato Etico Indipendente document n. 164/14, dated Nov. 21, 2014) who expressly considered the list of collaborators, anonymity of samples and the compliance with consent regulations of previous publications which included the same samples. The entire research was performed in accordance with relevant guidelines reported in the above mentioned document and those agreed upon by the scientific community.

### Experimental procedures

A detailed description of all methods is provided as Supplemental text. Briefly, a Roche Nimblegen custom array was used to capture a total of about 4.4 Mb, which excluded almost all the repetitive elements from the 22 X-degenerated blocks. A > 50× mean depth was obtained upon Illumina HiSeq. 2500 sequencing. Stringent filtering resulted in exclusion of one subject. Overall, 1079 variable positions (Supplemental Table 2) were detected out of 2,711,986 bp (Supplemental Table 3). Quality control included resequencing of six subjects (Supplemental Table 1), independently examined in a previous study^30^, and tree statistics (Supplemental Fig. 2). The transition/transversion ratio (Supplemental Table 5) and context dependency among the variants fitted genome-wide patterns^62^. The maximum parsimony (MP) tree was obtained with MEGA^63^ from the 1079 × 58 matrix of allele states (Fig. 1 and Supplemental Fig. 3). Node ages (Supplemental Table 7 and Supplemental Fig. 4) based on SNP diversity at 817 positions, were obtained with BEAST^64^ for the tree including also the Hg J individual “Kotias” (9.720 kya)^65^. The same program was used to obtain Bayesian Skyline Plots. Merging with the previous landmark studies was obtained by intersecting the covered positions and selecting Hg J carriers from the respective vcf files. We refrained from assembling a single tree for all subjects, as different filters applied in each study resulted in uncertain calls for some positions, some of which highly relevant for the Hg J topology. Genotyping of the core sample at 7 selected variable positions was by Sanger sequencing. The comparison of genotypes at the same 7 phylogeographically interesting markers was performed by scrutinizing each vcf file of the previous landmark studies separately.

Bam files have been deposited at the European Nucleotide Archive (https://www.ebi.ac.uk/ena) under study accession n. PRJEB25861 for 53 subjects, and sample accession n. ERS2065802-6 for subjects S206-S210, respectively.

## Electronic supplementary material

Supplemental text

Supplemental Tables
