# Supplementary material for: A finely resolved phylogeny of Y chromosome Hg J illuminates the processes of Phoenician and Greek colonizations in the Mediterranean
Source: Sci Rep. 2018 May 10;8:7465. doi: 10.1038/s41598-018-25912-9 (PMC5945646; doi:10.1038/s41598-018-25912-9)
Supplement: Supplementary file 1 — Supplemental text [file 41598_2018_25912_MOESM1_ESM.pdf]

A finely resolved phylogeny of Y chromosome Hg J illuminates the processes of Phoenician and Greek colonizations in the Mediterranean

Andrea Finocchio[1], Beniamino Trombetta[2], Francesco Messina[1], Eugenia D'Atanasio[2], Nejat Akar[3], Aphrodite Loutradis[4], Emmanuel I. Michalodimitrakis[5], Fulvio Cruciani[2,6], Andrea Novelletto[1*].

1. Department of Biology, University of Rome Tor Vergata, Rome, Italy

2. Dipartimento di Biologia e Biotecnologie "C. Darwin", Sapienza Università di Roma, Rome, Italy

3. Pediatrics Department, TOBB-Economy and Technology University Hospital, Ankara, Turkey

4. National Center for Thalassemias, Athens, Greece

5. Department of Forensic Sciences, University of Crete School of Medicine, Heraklion, Crete, Greece

6. Istituto di Biologia e Patologia Molecolari, CNR, Rome, Italy

Additional text: detailed methods

Targeted Next Generation Sequencing

Library preparation, targeting, sequencing and alignment were performed by BGI-Tech (Hong Kong). The targeted unique regions of the MSY were captured using a Roche Nimblegen custom capture array, composed of a set of 200 bp probes. The probes excluded almost all the repetitive elements from the 22 X-degenerated blocks, capturing a total of about 4.4 Mb. The captured regions were loaded onto an Illumina HiSeq 2500 platform to produce a >50× mean depth.

Low quality reads, contamination with adapters and repeated reads were discarded and the sequences of each subject were aligned to the human Y chromosome reference sequence (GRCh37/hg19) by means of the BWA (Burrows-Wheeler Aligner) software [1], generating an alignment file (.bam format)[2].

Data filtering and variant calling

The filtered regions were further refined with the removal of ~ 0.70 Mb within the repetitive elements (using the Repeat Masker and Simple Repeats tracks from the Table browser tool of the UCSC Genome Browser). Two blocks (chrY:21152521-21155078; chrY:28793241-28819317) with high average depth were also discarded, because this value could be indicative of chromosome rearrangements.

For each subject high quality positions were then retained [2] if base quality threshold >40, mapping quality threshold >30, a minimum depth of 3 and not higher than the 97.5 percentile of the depth distribution in the same subject. The positions passing these steps were intersected across subjects, arriving at 2,723,854 bp

Variant calls were obtained with both GATK [3] and FreeBayes [4], with haploid specific parameters and stringent criteria. Indels were removed. The remaining variants called differently by the two software were visually inspected with the Integrative Genome Viewer and rejected if any of these conditions were verified: 1) occurrence in short simple repeats, 2) involvement of multiple adjacent positions or 3) mean alternate fraction <75%. Exclusion of a variable position included removal of 100 bp on both sides, in order to minimize the impact on the overall density of variants per sequenced position. A final step consisted in the check, in each subject, of the equality of depth in the shared vs. private variants. One subject did not pass this step (t-test p=0.00013) and was

excluded. Variants called in all 58 subjects were excluded. The final dataset consisted of 1079 variable positions (Supplemental Table 2) out of 2,711,986 bp (Supplemental Table 3).

Quality controls

The number of private variants did not correlate with the overall sequencing depth across subjects (r^2 = 3E-6, n.s.).

For six subjects, targeted capture and sequencing was independently performed (Supplemental Table 1) in a previous study [5]. When the genotype calls of these subjects in the two studies were compared, no variant detected in ref.[5] was missed in the present study. Conversely, we scored an excess of 15 variants (Supplemental Table 4), yielding an overall rate of discrepant genotype calls of 0.98%. Three lines of evidence indicate that the majority of these can be considered bona fide variants. First, some of them were already recorded in dbSNP. Second, when previously detected (Isogg.org), the same variants were assigned to Hg J or some sub-haplogroup of it. Third, 11 of the 15 variants were reconfirmed in additional subjects among the 58 here studied (see branch assignment in Supplemental Table 2), and only 4 remained private and could not be further confirmed.

Overall, we observed a vast excess of transitions vs. transversions (Supplemental Table 5), in line with well-established genome-wide patterns[6]. We further explored the dependency of variant type on the nucleotide context [7], by downloading from the reference sequence the trinucleotides centred on each of our 1079 variable positions (Supplemental Table 2). The observations in the resulting 64 contexts x 3 possible substitutions contingency table were compared with expectations conditioned on the overall rates of the 12 possible substitution types by a 1 d.f. Chi-square test, and the results summarized in a Q-Q plot (Supplemental Fig. 2A). Overall, we counted 6 5'-TCC-3' -> 5'-TTC-3' and 13 5'-GGA-3' -> 5'-GAA-3' transitions, respectively. The corresponding aggregate frequency was 0.017, in line with the Y chromosome data [7].

SNP and STR genotyping

Genotyping of additional subjects at selected variable positions was by Sanger sequencing of PCR products obtained with primers designed with PrimerBLAST (https://www.ncbi.nlm.nih.gov/tools/primer-blast/index.cgi?LINK_LOC=BlastHome) on the 2000 bp centred on the target position (Supplemental Table 6). For each of these SNP, only carriers of the lineage candidate to harbour the derived allele at the target positions were genotyped.

Genotyping at seven microsatellite loci (YCAIIa, YCAIIb, DYS19, DYS390, DYS391, DYS392 and DYS393) was by PCR, in the presence of fluorescently labelled primers [8] and separation in an automated sequencer. PCR fragment sizes were transformed in repeat sizes by comparison with sequenced standards.

Tree construction and statistics

The maximum parsimony (MP) tree was obtained with MEGA [9] from the 1079 x 58 matrix of allele states (Figure 1 and Supplemental Figure 3). The MP tree inferred a single recurrent mutational event at a CpG dinucleotide (Y:16610308) (Supplemental Table 2). Topology and branch lengths were further verified by constructing a median-joining network with the program NETWORK [10] on the same matrix. This was also used to calculate node ages with the rho method [11].

The Branch Length Ratio test [7] was obtained by calculating the length of both descending branches from the MRCA of all possible pairs of tree tips (subjects), with the R package "ape" [12]. The departure from the null, equal length hypothesis, was evaluated by a 1 d.f. Chi-square test, and the results summarized in a Q-Q plot (Supplemental Fig. 2B).

NETWORK was also used to represent the relationships between 7-STR haplotypes within SNP-defined clades, weighting each STR locus according to the inverse of its repeat variance and modelling SNPs as additional loci with weight = 99.

Dating

Node ages based on SNP diversity (Supplemental Table 7) were obtained with BEAST [13] for the tree including also the Hg J individual "Kotias" [14]. This was given an age of sampling of 9,720 years ago, i.e. the midpoint of the range in calibrated years (Supplemental Figure 4). A lognormal relaxed clock model was used, with all parameters as in ref. [15]. Two runs of 10E6 steps were performed (20% burnin), and combined.

With this method we obtained an estimated mutation rate of 8.88E-10 events/bp/year (95% C.I. 5.50E-10 - 12.2E-10). When projected onto the 2.7 Mb resequenced in the 58 modern subjects, this translated into a rate of one mutation every 415 years, which was used to obtain node ages with the rho method (Supplemental Table 7).

Ages of nodes to which SNP and STR genotyping was applied were also obtained with BATWING [16]. Priors for STR mutation rates were obtained from father-son trasmissions

(https://yhrd.org/); gamma(2,1000) was used for YCAIIa and YCAIIb. SNP data were included in the input files, when appropriate, with no inference on the SNP mutation rate. Ages of the SNP event were obtained from the descendant and ancestral STR node times of the branch on which the mutation occurred (Supplemental Table 7).

Demographic reconstructions

Bayesian Skyline Plots [17] (Supplemental Figure 5) were obtained with BEAST. For this analysis we used the whole tree as well as the 5 clades devoid of nested subclades also used in the selection process. This strategy aimed at avoiding over/down representation of downstream lineages (e.g. J2a-M92 within J2a-M67). A lognormal relaxed clock and a mutation rate of 8.8E-10 (see above) were used, with 10 and 4 groups for the whole tree and internal lineages, respectively.

Merging with other datasets

Merging with the previous landmark studies was obtained by selecting Hg J carriers from the respective vcf files. The lists of resequenced positions (bed files) were intersected with those covered here with bedtools, to obtain distinct reduced vcf files from each of the external sources. We refrained from assembling a single tree for all subjects, as different filters applied in each study resulted in uncertain calls for some positions, some of which highly relevant for the Hg J topology. The comparison of genotypes at phylogeographically interesting markers was performed by scrutinizing each vcf file separately.

For the ancient specimen "Kotias" [14], a bed file was initially created, containing positions with a minimal coverage of 4. A vcf file was obtained with base quality threshold >30, mapping quality threshold >20 and a mean alternate fraction of 0.87. Visual inspection was applied to recover positions with very low coverage. Four variants were recovered, displaying 1 and 3 ref/alt reads, respectively. The resulting vcf was merged with ours (see above), limited to shared positions. The final dataset consisted in 817 variable positions extracted from 2,043,333 bp (Supplemental Figure 4).

Additional analyses

A Venn diagram for the sharing of variable positions across studies (Supplemental Figure 6) was obtained with the tool available at http://bioinformatics.psb.ugent.be.

Maps were drawn with scripts in R[18] and sampling locations superimposed based on their long-lat coordinates (Figure 2 and Supplemental Figure 7). Centroids were obtained by calculating Mercator coordinates, taking averages for long and lat, and re-transforming in degree coordinates. A test for the spatial shift of the centroid of the derived alleles was performed by simulation: the 95% C.I. for the centroids of ancestral alleles were obtained by drawing 1000 random samples of the same size as the n. of derived alleles, calculating their centroids and the ellipse embracing 95% of them. This procedure was omitted for rs760148062, as the number of ancestral and derived alleles was similar, approaching the extraction of 1000 replicas of the same centroid. Pairwise sampling distances were obtained with the R function rdist.earth separately for carriers of ancestral and derived alleles. Their equality was tested using the t-test.

References

1 Li, H. & Durbin, R. Fast and accurate short read alignment with Burrows-Wheeler transform. *Bioinformatics* **25**, 1754-1760 (2009).

2 Li, H. *et al.* The Sequence Alignment/Map format and SAMtools. *Bioinformatics* **25**, 2078-2079 (2009).

3 McKenna, A. *et al.* The Genome Analysis Toolkit: A MapReduce framework for analyzing next-generation DNA sequencing data. *Genome Res* **20**, 1297-1303 (2010).

4 Garrison, E. & Marth, G. Haplotype-based variant detection from short-read sequencing. *arXiv* **1207.3907**, (2012).

5 Hallast, P. *et al.* The Y-chromosome tree bursts into leaf: 13,000 high confidence SNPs covering the majority of known clades. *Mol Biol Evol* **32**, 661-673 (2015).

6 Ségurel, L.,Wyman, M. J. & Przeworski, M. Determinants of mutation rate variation in the human germline. *Annu Rev Genomics Hum Genet* **15**, 47-70 (2014).

7 Harris, K. Evidence for recent, population-specific evolution of the human mutation rate. *Proc Natl Acad Sci U S A* **112**, 3439-44 (2015).

8 Butler, J. M. *et al.* A novel multiplex for simultaneous amplification of 20 Y chromosome STR markers. *Forensic Sci Int* **129**, 10-24 (2002).

9 Tamura, K. *et al.* MEGA5: Molecular Evolutionary Genetics Analysis using maximum likelihood, evolutionary distance, and maximum parsimony methods. *Mol Biol Evol* **28**, 2731-2739 (2011).

10 Bandelt, H. J.,Forster, P. & Rohl, A. Median-joining networks for inferring intraspecific phylogenies. *Mol Biol Evol* **16**, 37-48 (1999).

11 Forster, P.,Harding, R.,Torroni, A. & Bandelt, H. J. Origin and evolution of Native American mtDNA variation: a reappraisal. *Am J Hum Genet* **59**, 935-945 (1996).

[12] Popescu, A.-A.,Huber, K. T. & Paradis, E. ape 3.0: new tools for distance based phylogenetics and evolutionary analysis in R. *Bioinformatics* **28**, 1536-1537 (2012).

[13] Drummond, A. J. & Rambaut, A. BEAST: Bayesian evolutionary analysis by sampling trees. *BMC Evol Biol* **7**, 214 (2007).

[14] Jones, E. R. *et al.* Upper Palaeolithic genomes reveal deep roots of modern Eurasians *Nat Commun* **6**, 8912; 10.1038/ncomms9912 (2015).

[15] Trombetta, B. *et al.* Regional differences in the accumulation of SNPs on the male-specific portion of the human Y chromosome replicate autosomal patterns: implications for genetic dating. *PLoS ONE* **10**, e0134646 (2015).

[16] Wilson, I. J. & Balding, D. J. Genealogical inference from microsatellite data. *Genetics* **150**, 499-510 (1998).

[17] Drummond, A. J.,Rambaut, A.,Shapiro, B. & Pybus, O. G. Bayesian coalescent inference of past population dynamics from molecular sequences. *Mol Biol Evol* **22**, 1185-1192 (2005).

[18] R Core Team R: A Language and Environment for Statistical Computing. Vienna, Austria (2014).

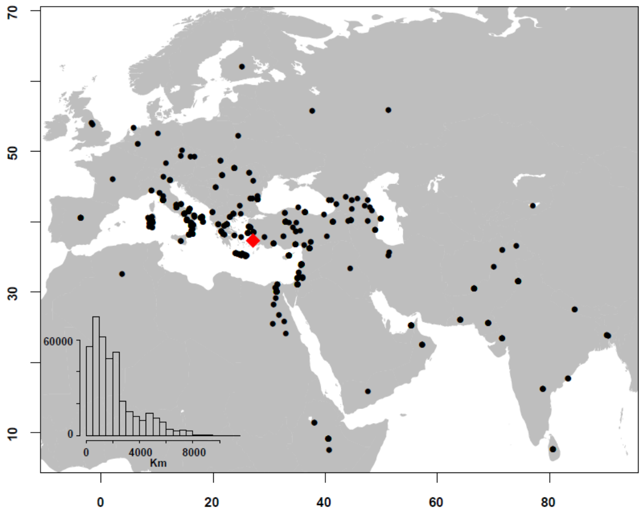

Supplemental Figure 1. Map of sampling locations for 893 carriers of Hg J chromosomes. Coastlines were drawn with the R packages[18] "map" and "mapproj" v. 3.1.3 (https://cran.r-project.org/web/packages/mapproj/index.html), and additional features added with default functions. Dot size is not proportional to the number of subjects for each location. The red lozenge indicates the centroid. In the inset: histogram of pairwise sampling distances.

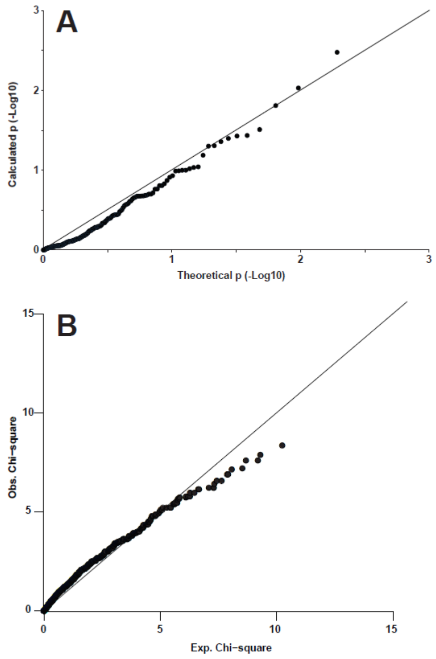

Supplemental Figure 2. QQ plots of Chi-square statistics used as quality controls. A) Test of the dependency of substitution type on the nucleotide context; B) Branch length ratio test.

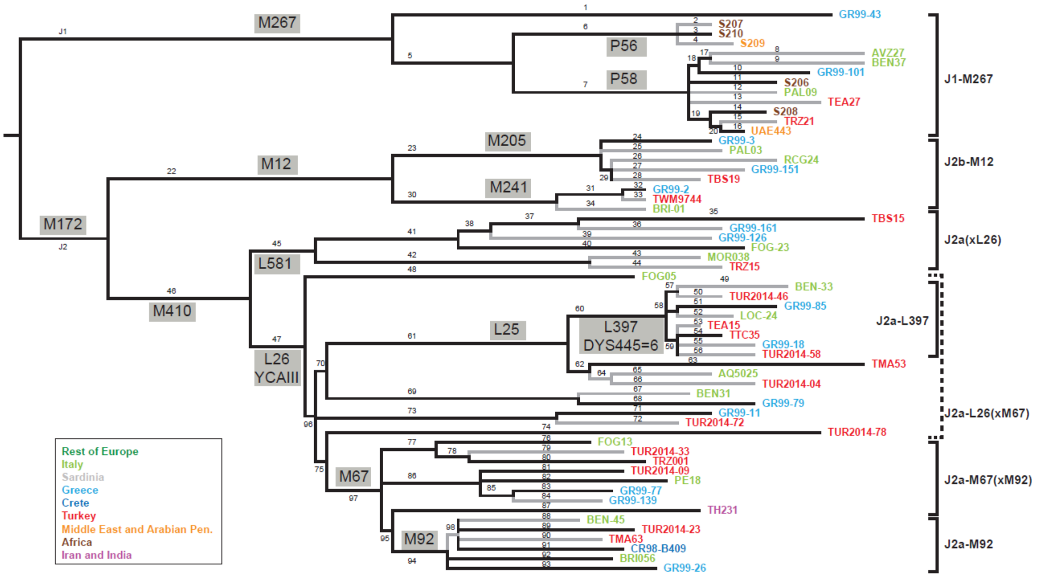

Supplemental Figure 3. Same tree as in Figure 1, with individuals identified by their labels. Branches are numbered sequentially as in Supplemental Table 2. In grey: branches for which 100% of SNPs were not recorded in dbSNP 147. Markers discussed in the text are shown next to the branches where they occur, in grey boxes.

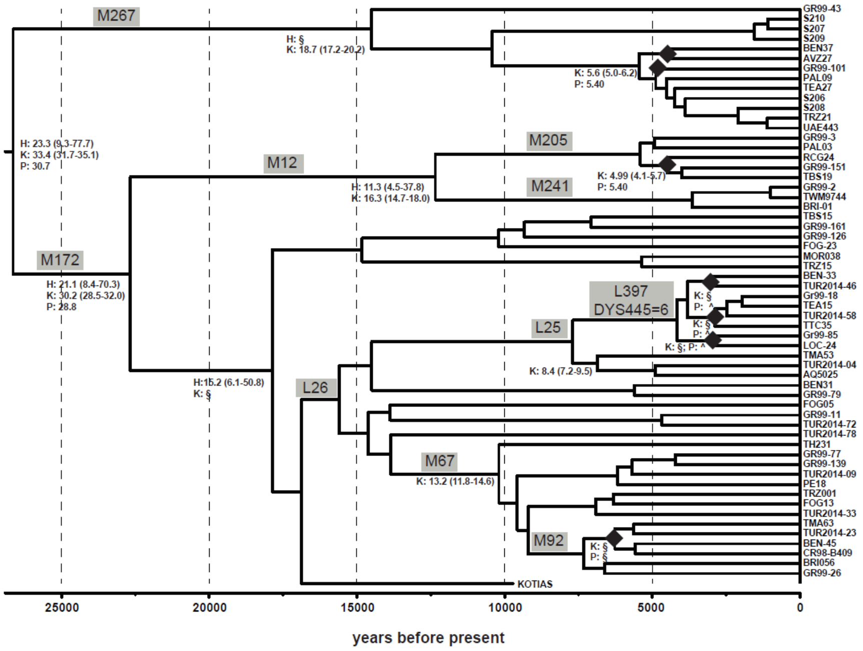

Supplemental Figure 4. Bayesian tree obtained with BEAST, calibrated with the ancient Hg J subject "Kotias" (Jones et al. 2015). The original subjects' labels are reported at tips. Note that the program resolved all multifurcations of the MP tree into sequential bifurcations. Lozenges indicate the same nodes as in Figure 1. For immediately corresponding nodes, the ages (and C.I. in parentheses) obtained in three previous works are reported: H = Hallast et al. (2015); K = Karmin et al. (2015); P = Poznik et al. (2016). The following symbols are also used: § = node not comparable across studies; ^ = node not dated in the reported study.

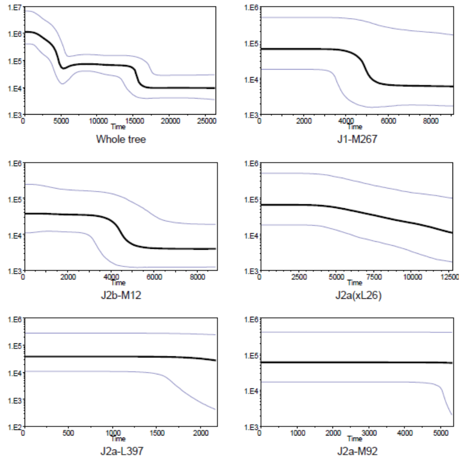

Supplemental Figure 5. Bayesian Skyline Plots obtained in the whole tree and the indicated subclades (see Supplemental text). Note the different time scales across plots.

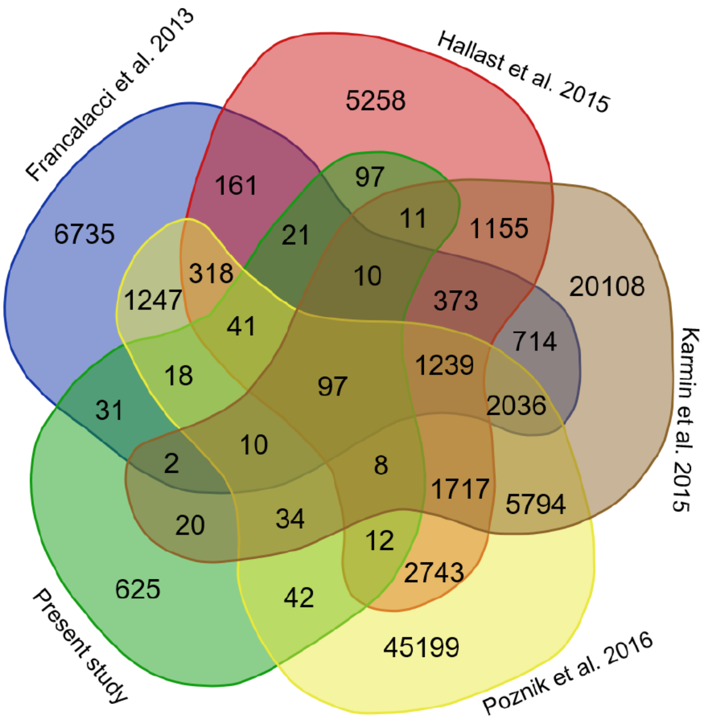

Supplemental Figure 6. Venn diagram showing the number of variants shared among 5 Y chromosome resequencing studies.

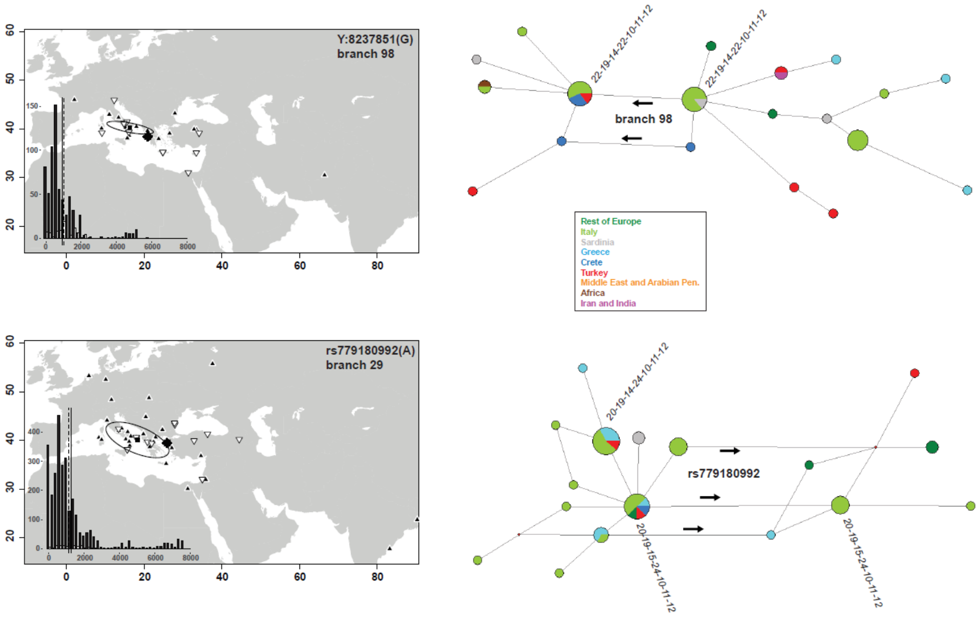

Supplemental Figure 7. Maps of sampling locations for the carriers of the derived allele (white triangle point down) at the indicated SNP vs carriers of the ancestral allele (black triangle point-up), conditioned on identical genotype at the same most terminal marker. Coastlines were drawn with the R packages[18] "map" and "mapproj" v. 3.1.3 (https://cran.r-project.org/web/packages/mapproj/index.html), and additional features added with default functions. The star triangle indicates the centroid of derived alleles. The solid square indicates the centroid of ancestral alleles, with its 95% C.I. (ellipse). In the insets: distributions of the pairwise sampling distances (in Km) for the carriers of the ancestral (black) and derived (white) allele, with solid and dashed lines indicating the respective averages. At right: median joining network of 7-STR haplotypes and SNPs in the same groups, with sectors coloured according to sampling location. Haplotype structure is detailed for some nodes, in the order YCA2a-YCA2b-DYS19-DYS390-DYS391-DYS392-DYS393 (in italics).
